# Vaccine-preventable diseases.

**DOI:** 10.3201/eid0403.980314

**Published:** 1998

**Authors:** A C Mawle

**Affiliations:** Centers for Disease Control and Prevention, Atlanta, Georgia, USA.


### Vaccine-Preventable Diseases

**Alison C. Mawle**
Centers for Disease Control and Prevention, Atlanta, Georgia, USA

The panel addressed four main areas of vaccine development and use: diseases of public health importance for which no vaccine is available; diseases for which an existing licensed vaccine is not optimal and alternative vaccines are under development; diseases for which a vaccine exists but is not being used optimally; and diseases requiring vaccines of specialized or limited use, such as those needed for controlling outbreaks or for military use. The specific diseases chosen to illustrate each area were malaria, influenza, meningitis, and filovirus infections (Ebola and Marburg), respectively.

Lee Hall, National Institute of Allergy and Infectious Diseases, addressed the basic challenges in developing a malaria vaccine. The historical norm of vaccine development has been an empirical process; in contrast, modern vaccines take advantage of basic knowledge of the organism and of the immune response to it. In vaccine development, certain elements are prerequisite: demonstrable protective immunity and intimate knowledge of the organism's life cycle, including the DNA sequence. Vaccine development has three potential goals: prevent infection, prevent disease, and prevent transmission. A successful vaccine may address any or all these areas; for malaria, a vaccine able to perform any of these would have a significant impact. Vaccine development, often thought of as a flow, is in fact an iterative process; it addresses scientific, technical, manufacturing, and clinical issues and is affected by economic, political, and social issues usually outside the scientific sphere of influence. Several downstream gaps in vaccine development include resource limitation, lack of standardization, and problems in clinical and industrial interest.

Claude Hannoun, Institut Pasteur, addressed problems in influenza vaccine development. Influenza, the quintessential emerging infectious disease, needs a new vaccine each year to protect against the predominant strains. The disease poses additional challenges; one is the need for vaccine against a potential pandemic strain, particularly a pandemic strain whose epidemiologic characteristics are different from those of usual strains (e.g., the 1918 strain killed young adults). Vaccine production problems pose another challenge. Identifying an appropriate seed strain can delay initiation of production for 4 to 6 months after the need for a vaccine has been identified, and growing sufficient quantities of vaccine is difficult. The issue of an appropriate vaccine regimen (one dose or two) was also addressed. The many vaccination issues involved in a pandemic situation make the adoption of a credible pandemic plan imperative. The emergence of the H5N1 strain in Hong Kong has underlined this imperative.

Brad Perkins, Centers for Disease Control and Prevention, presented the challenge of the African meningitis belt, where periodic large epidemics affect approximately 1% of the population with a 10% death rate. Since the current vaccine does not confer lasting protection, prediction of these epidemics can trigger vaccination campaigns to prevent deaths. A model using an epidemic threshold of 15 cases per 100,000 demonstrated potential lives saved. Obstacles to the use of the vaccine include inadequate surveillance, high cost of the vaccine, inadequate delivery systems, and inadequate vaccine supply.

Vaccines for agents such as Marburg and Ebola were discussed by Alan Schmaljohn, U.S. Army Medical Research Institute of Infectious Diseases. Limited-use vaccines do not have a global market but are potentially important against the threat of biological weapons or epidemics. These vaccines have unique problems, such as inadequate efficacy testing (since there is no disease-endemic area) and high production costs (since there is no target population).

All the presentations addressed public policy issues: who will use the vaccine and under what circumstances, what is the time frame for vaccine development (short-term versus long-term), what is the cost of a vaccine (who bears the brunt of development costs), how are these costs recouped, and what is the role of partnerships in determining vaccine need and use.

